# The Relationship between Bone Mineral Densitometry and Visceral Adiposity Index in Postmenopausal Women

**DOI:** 10.1055/s-0043-1764497

**Published:** 2023-03-28

**Authors:** Halis Elmas, Cevdet Duran, Mustafa Can, Ismet Tolu, Ibrahim Guney

**Affiliations:** 1 Department of Internal Medicine, Reyhanli State Hospital, Hatay, Turkey; 2 Division of Endocrinology and Metabolism, Department of Internal Medicine, Usak University School of Medicine, Usak, Turkey; 3 Division of Endocrinology and Metabolism, Necmettin Erbakan University Meram School of Medicine, Konya, Turkey; 4 Department of Radiology, University of Health Sciences, Konya City Hospital, Konya, Turkey; 5 University of Health Sciences, Konya City Hospital, Konya, Turkey

**Keywords:** densidade mineral óssea, absortometria de raio X de dupla energia, osteoporose pós-menopausa, obesidade, índice de adiposidade visceral, bone mineral density, dual-energy X-ray absorptiometry, postmenopausal osteoporosis, obesity, visceral adiposity index

## Abstract

**Objective** It was aimed to compare visceral adiposity index (VAI) levels in patients with normal bone mineral density (BMD), osteopenia, and osteoporosis.

**Methods** One hundred twenty postmenopausal women (40 with normal BMD, 40 with osteopenia, and 40 with osteoporosis) between the ages of 50 to 70 years were included in the study. For females, the VAI was calculated using the formula (waist circumference [WC]/[36.58 + (1.89 x body mass index (BMI))]) x (1.52/High-density lipoprotein [HDL]-cholesterol [mmol/L]) x (triglyceride [TG]/0.81 [mmol/L]).

**Results** The time of menopause from the beginning was similar in all groups. Waist circumference was found to be higher in those with normal BMD than in the osteopenic and osteoporotic groups ( *p* = 0.018 and *p* < 0.001, respectively), and it was also higher in the osteopenic group than in the osteoporotic group ( *p* = 0.003). Height and body weight, BMI, blood pressure, insulin, glucose, HDL-cholesterol, and homeostasis model assessment-insulin resistance (HOMA-IR) levels were similar in all groups. Triglyceride levels were found to be higher in the normal BMD group, compared with the osteoporotic group ( *p* = 0.005). The level of VAI was detected as higher in those with normal BMD, compared with the women with osteoporosis ( *p* = 0.002). Additionally, the correlation analysis showed a positive correlation between dual-energy X-ray absorptiometry (DXA) spine *T* -scores, WC, VAI, and a negative correlation between DXA spine *T* -scores and age.

**Conclusion** In our study, we found higher VAI levels in those with normal BMD, compared with women with osteoporosis. We consider that further studies with a larger sample size will be beneficial in elucidating the entity.

## Resumo

**Objetivo** O objetivo foi comparar os níveis de índice de adiposidade visceral (IVA) em pacientes com densidade mineral óssea (DMO) normal osteopenia e osteoporose.

**Métodos** Cento e vinte mulheres na pós-menopausa (40 com DMO normal 40 com osteopenia e 40 com osteoporose) com idades entre 50 e 70 anos foram incluídas no estudo. Para o sexo feminino o VAI foi calculado pela fórmula (circunferência da cintura [CC]/[36 58 + (1 89 x índice de massa corporal (IMC))]) x (1 52/lipoproteína de alta densidade [HDL]-colesterol [mmol/L] ) x (triglicerídeo [TG]/0 81 [mmol/L]).

**Resultados** O tempo de menopausa desde o início foi semelhante em todos os grupos. A circunferência da cintura foi maior naqueles com DMO normal do que nos grupos osteopênicos e osteoporóticos (p = 0 018 e p < 0 001 respectivamente) e também foi maior no grupo osteopênico do que no grupo osteoporótico (p = 0 003) . Altura e peso corporal IMC pressão arterial insulina glicose HDL-colesterol e os níveis de avaliação do modelo de homeostase-resistência à insulina (HOMA-IR) foram semelhantes em todos os grupos. Os níveis de triglicerídeos foram maiores no grupo DMO normal em comparação com o grupo osteoporótico (p = 0 005). O nível de VAI foi detectado como maior naquelas com DMO normal em comparação com as mulheres com osteoporose (p = 0 002). Além disso a análise de correlação mostrou uma correlação positiva entre a absorciometria de raios-X de dupla energia (DXA) nas pontuações T da coluna CC VAI e uma correlação negativa entre as pontuações T da coluna DXA e a idade.

**Conclusão** Em nosso estudo encontramos níveis mais elevados de VAI naquelas com DMO normal em comparação com mulheres com osteoporose. Consideramos que novos estudos com maior tamanho amostral serão benéficos na elucidação da entidade.

## Introduction

Osteoporosis is a disease resulting in an increase in bone fragility and fracture tendency due to the low bone mass and deterioration of the microarchitecture of bone tissue. 1 Postmenopausal osteoporosis is a common disease and seen among women at the age of ≥ 50 years; the measurement of bone mineral density (BMD) is the most important test used in the diagnosis of osteoporosis. 2

The determination of the amount of body fat is important for the accurate diagnosis of obesity, and there are various direct and indirect methods used for measuring body fat. 3 The regional distribution of adipose tissue is an important indicator of the degree of metabolic derangement. 4 5 The storage of adipose tissue around the visceral organs carries a higher risk of metabolic disorders, such as dyslipidemia and hyperinsulinemia and is more closely associated with those diseases, compared with the adipose tissue stored under the skin. The visceral adiposity index (VAI) calculated using such values as body mass index (BMI), waist circumference (WC), triglyceride (TG), and high-density lipoprotein (HDL)-cholesterol has been demonstrated to reflect the distribution of visceral fat and insulin resistance (IR) very well. 6 Visceral adiposity index has also been reported to be an indicator of adipose tissue dysfunction and abnormal fat distribution and to be associated with cardiometabolic risk and subclinical inflammation. 7

Obesity and osteoporosis are well known to be diseases related to each other but also affected by genetic and environmental factors. 8 The studies conducted so far suggest that obesity has a protective effect on osteoporosis. 9 10 11 12 13 14 15 16 17 Based on the literature, many researchers have investigated the mechanism whereby obesity affects osteoporosis. In general conclusion, obesity has a protective effect against osteoporosis. In various studies, obesity has been stated to inhibit bone resorption by increasing estrogen levels and stimulating osteoclast apoptosis; in addition, total cholesterol (TC), low-density lipoprotein (LDL)-cholesterol, and TG levels are lower in those with vertebral fractures, and each 1 mg/dL increase in TC levels has reduced the risk of vertebral fractures by 2.2%, and, thus, many known or unknown hormonal and mechanical factors have been protective against osteoporosis in obesity. 18 19 On the other hand, studies are reporting that obesity has no, or negative, effects on the development of osteoporosis. 20 21 22 23 It has been reported that the release of many inflammatory factors by the visceral adipose tissue (VAT), especially compared with the subcutaneous adipose tissue, increases bone resorption in patients with osteoporosis. 24 In another study, however, it is emphasized that the subcutaneous tissue is the main fat tissue of the body, and such hormones as leptin released from these areas increase bone formation. 25

To the best of our knowledge, there is no study in the literature investigating the relationship between BMD and the levels of VAI. The present study, therefore, aimed to investigate the levels of VAI in postmenopausal women with normal BMD, osteopenia, osteoporosis, and the association between the levels of VAI and the lumbar spine *T* -scores of BMD.

## Methods

The present study was performed in the Division of Endocrinology and Metabolism of the department of internal medicine in the Konya Training and Research Hospital between January to June 2017. The study was performed under the principles of the 1964 declaration of Helsinki and its later amendments, and the study protocol was also approved by the ethical board of Necmettin Erbakan University. According to BMD scores evaluated through DXA, 120 patients (40 with normal BMD, 40 with osteopenia, and 40 with osteoporosis) between > 50 and < 70 years of age who were menopausal for at least 1 year at the time of the enrollment were included in the study. The participants were put into 3 age- and BMI-matched groups with normal BMD, osteopenia, and osteoporosis. The women < 50 and > 70 years of age with a history of premature or surgical menopause, those having any disease-causing secondary osteoporosis and known celiac disease or any disease leading to malabsorption such as pancreatic insufficiency, those undergoing bariatric surgery or any intra-abdominal surgery that may cause malabsorption, those receiving any previous treatment due to osteoporosis, those administered with hormone replacement therapy containing estrogen at that time or previously in the postmenopausal period, those given previously lipid-lowering medications at least for 6 months, those with active infection foci determined in the physical examination, and those with any known rheumatologic disease, any type of cancers or chronic infections, and liver and kidney failures, and those smoking cigarettes and/or consuming alcoholic beverages were excluded from the study. The patient's age, weight, height, BMI, WC, and systolic (SBP) and diastolic blood pressure (DBP) were recorded in the study files. At once, blood samples were obtained from the same veins of each patient following 12 hours of fasting to measure TG, HDL-cholesterol, glucose, and insulin, and separated from the serums by centrifugation at a speed of 3,600 rpm for 10 minutes until the measurements and the serums were frozen at -80°C. The bone mineral densitometry readings of the patients were obtained from the measurements recorded through the DXA method using the Hologic brand QDR 4500 W device (Hologic Inc., Bedford, MA, USA) in the bone densitometer unit of the radiology department. The total BMD changes of the lumbar vertebra and femur were considered through the *T* -scores. Based on the literature, those with BMD *T* -scores of 2.5 standard deviations (SDs) or more below the average BMD of the young adult reference population at any site were detected as osteoporosis, on condition that other causes of low BMD were ruled out (such as osteomalacia); a *T* -score of 1 to 2.5 SD below the average of the young adult population was termed low bone mass (osteopenia), and the normal bone density was defined as the value in +1 SD of the average in the young adult reference individuals. 1

While the levels of glucose were measured with the hexokinase method using the Olympus AU 5800 (Beckman Coulter Inc., Brea, CA, USA), insulin levels ([NR], 6–27 μlU/mL) were measured via the chemiluminescence method using the Immulite 2000 device (Siemens Healthcare Diagnostics, Germany). HDL-cholesterol levels ([NR], 40–90 mg/dL) were also measured with the immune reaction (antigen-antibody complex) through an Olympus AU 5800 device (Beckman Coulter Inc), and the levels of TG ([NR], 60–150 mg/dL) were measured using a routine enzymatic method with the auto analyzer of Olympus AU 5800 device (Beckman Coulter Inc). However, BMI was calculated using body weight (kg)/height 2 ([meter [m] 2 ), and VAI was also calculated with the help of (WC/[36.58 + (1.89 x BMI)]) x (1.52/HDL [mmol/L] x TG/0.81[mmol/L]) formula. 6 The homeostasis model assessment-insulin resistance (HOMA-IR) index was calculated based on the formula (fasting glucose levels [mg/dL] x fasting insulin levels [μU/mL])/405. 26

Data analyses were performed with the IBM SPSS Statistics for Windows, Version 20.0 (IBM Corp., Armonk, NY, USA). Whether the distribution of continuous variables was close to the normal values was investigated using the Kolmogorov-Smirnov test. While the student *t* -test was used to compare the data with normal distribution between both groups, the results were given as mean ± SD. The Mann-Whitney-U test was utilized to compare the data without normal distribution between the two groups, and the results were presented as median (minimum:maximum). The Spearman correlation coefficient was used to analyze the relationship between continuous variables, and the regression analysis was performed to identify the independent risk factors for osteoporosis. A *p* -value lower than 0.05 was considered statistically significant.

## Results

**Fig. 1:**
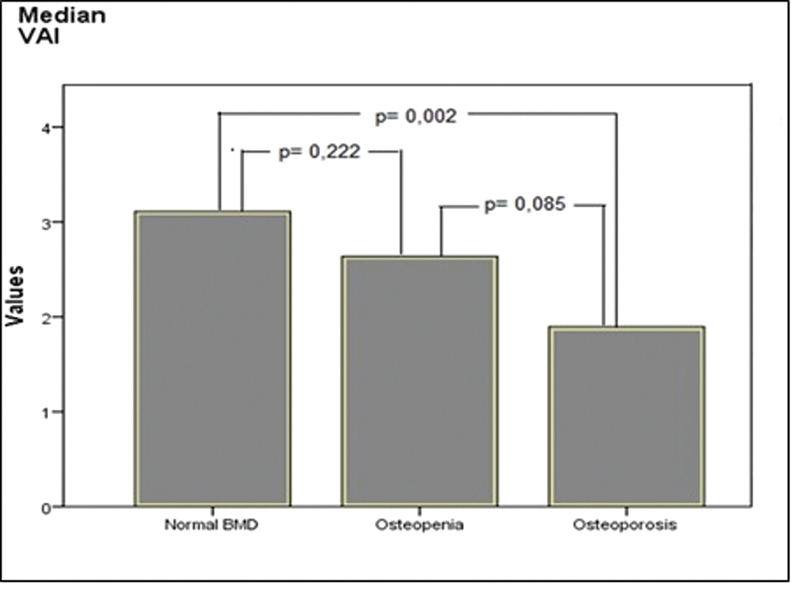
Comparison of normal BMD, osteopenic, and osteoporotic groups in terms of visceral adiposity index (VAI). BMD: bone mineral densitometry.

A total of 120 postmenopausal women aged between 50 and 70 years were included in the study. Based on the DXA lumbar spine *T* -score results, 3 age-matched groups were constituted of 40 women with normal BMD, 40 with osteopenia, and 40 with osteoporosis. No significant difference was determined between the three groups in terms of the measurements of body height, weight, BMI, SBP and DBP, insulin, glucose, HDL-cholesterol, and HOMA-IR levels ( Table 1 ). The time of menopause from the beginning was similar in all groups. Waist circumference was found to be higher in the normal BMD group, compared with the osteopenic and osteoporotic groups ( *p* = 0.018 and *p* < 0.001, respectively); however, when comparing the osteopenic and the osteoporotic groups, WC was found to be higher in the osteopenic group ( *p* = 0,003). The levels of TG were higher in those with normal BMD values compared with the osteoporotic group ( *p* = 0.005) ( Table 1 ). The level of VAI was also found to be higher in patients with normal BMD values, compared with those with osteoporosis ( *p* = 0.002) ( Table 1 and Fig. 1 ).

**Table 1:** Demographic characteristics of the study populations

	Women with normal BMD	Women with osteopenia	Women with osteoporosis	p1	p2	p3
Age (years)	58 (50:70)	61 (51:70)	61.50 (52:70)	0.182	0.060	0.563
Time of menopause (years)	11.5 (3:24)	12 (3:26)	12 (2:21)	0.851	0.721	0.855
Height (cm)	154.5 (145:170)	154 (142:170)	152.5 (140:165)	0.973	0.116	0.102
Weight (kg)	81.5 (45:120)	78 (54:106)	75 (50:93)	0.419	0.009	0.033
BMI (kg/m ^2^ )	33.7 ± 6.4	32.80 ± 4.50	31.7 ± 4.80	0.488	0.122	0.289
WC (cm)	102.1 ± 13.9	94.9 ± 12.6	85.9 ± 13.4	0.018	< 0.001	0.003
SBP (mmHg)	134 (100:168)	134 (114:164)	127 (114:151)	0.760	0.216	0.272
DBP (mmHg)	78 (60:93)	80 (61:94)	75 (62:98)	0.257	0.693	0.106
Lumbar spine DXA T-Score	-0.30 (-0.9:2.8)	-1.8 (-1:-2.4)	-3.0 (-2.6:-4)	< 0.001	< 0.001	< 0.001
Glucose (mg/dL)	98 (80:290)	94.5 (73:271)	98 (75:440)	0.436	0.981	0.541
Insulin (µU/mL)	13.5 (2.6:99)	14.4 (2:84.9)	11.3 (2:56.3)	0.587	0.573	0.290
HDL-cholesterol (mg/dL)	50.4 ± 9.5	51.4 ± 12.1	52.2 ± 12.2	0.668	0.464	0.784
TG (mg/dL)	184 (82:590)	171 (64:397)	133 (55:578)	0.405	0.005	0.078
HOMA-IR	3.2 (0.6:38.9)	3.7 (0.4:27.7)	2.9 (0.4:20.2)	0.862	0.564	0.413
VAI	3.1 (1:13.1)	2.6 (0.8:8.1)	1.9 (0.6:6.9)	0.222	0.002	0.085

Abbreviations: BMD, bone mineral densitometry; BMI, body mass index; DBP, diastolic blood pressure; DXA, dual-energy X-ray absorptiometry; HDL-cholesterol, high-density lipoprotein cholesterol; HOMA-IR, homeostasis model assessment-insulin resistance; p1, women with normal BMD *vs* women with osteopenia; p2, women with normal BMD *vs* women with osteoporosis; p3, women with osteopenia *vs* women with osteoporosis; SBP, systolic blood pressure; TG, triglyceride; VAI, visceral adiposity index; WC, waist circumference.

Based on the results of the correlation analysis, a positive correlation was determined between DXA lumbar spine *T* -scores, and WC and VAI, and a negative correlation was found between DXA lumbar spine *T* -scores and age ( Table 2 ). Based on the logistic regression analysis, it was found that each 1-year increase in the time of menopause causes a 0.096-decrease in the *T* -score of DXA lumbar spine ( *p* = 0.004), and a 1-cm increase in WC leads to a 0.037-increase in the *T* -score of DXA lumbar spine ( *p* < 0.001) ( Table 3 ).

**Table 2:** Correlation analysis* between dual-energy X-ray absorptiometry lumbar spine *T-score* and some parameters

	r	*p*
Age (years)	-0.181	0.047
WC	0.442	< 0.001
VAI	0.199	0.030

Abbreviations: VAI, visceral adiposity index; WC, waist circumference.

*The Spearman correlation test was performed for the correlation analysis.

**Table 3:** Regression analysis for the factors affecting bone mineral densitometry

Model	Unstandardized coefficients		Standardized coefficients	t	Sig.
	B	Std. Error	Beta		
(Constant)	-0.695	1.761		-0.395	0.694
Menopause age	-0.096	0.032	-0.246	-2.976	0.004
WC	0.037	0.008	0.399	4.877	< 0.001
TG	0.001	0.001	0.099	1.193	0.235

Abbreviations: TG, triglyceride; WC, waist circumference.

## Discussion

This is the first study to investigate the relationship between VAI and BMD, and, in the present study, the VAI levels were found to be lower in women with osteoporosis compared with those with normal BMD values. In addition, the increase in WC and VAI levels was also shown to have a positive protective effect on the *T* -score results of the DXA lumbar spine.

It is well-known that obesity and osteoporosis are diseases having mutual effects on each other and are affected by genetic and environmental factors. 8 Several previous studies suggest that obesity has a protective effect on osteoporosis. 9 10 11 12 13 14 15 16 17 18 19 Although it has been stated that such conditions as increased estrogen levels or hyperlipidemia accompanying obesity, or many unknown hormonal and mechanical factors, have positive effects on bones and are protective against the development of osteoporosis, 18 19 there are also studies reporting that obesity has no or negative effects on the development of osteoporosis. 20 21 22 23 While subcutaneous adipose tissue has protective effects on bones in terms of osteoporosis development, it is known that VAT, which is more active in terms of inflammatory factor release, has negative effects on the development of osteoporosis. 24 25 In a study in which 1,005 postmenopausal patients < 75 years of age and admitted to the Fracture Liaison Service with low trauma complaints were evaluated by Premaor et al., 13 59.1% of the obese participants (defined as BMI ≥ 35 kg/m ^2^ ) and 73.1% of the morbidly obese participants were stated to have normal BMD values, and the incidence rates of osteoporosis were reported to be 11.7% and 4.5%, respectively. In the same study, there was also a negative correlation between the hip *T* -score and age, but a positive correlation between the hip *T* -score and BMI. However, in the study performed by Ong et al. 14 with 4,288 patients monitored due to low-trauma fractures, the rates of osteoporosis were found to be 13.4, 24.9, and 40.4% in obese and overweight patients, and those with normal-weight limits, respectively. In another study performed with 2,631 patients by Zillikens et al., 12 it was emphasized that a positive relationship between fat distribution and BMD was shown only in patients with the android type (visceral) of obesity, but not in those with the gynoid type of obesity. In a study 23 evaluating adipose tissue in pre- and postmenopausal women with DXA, it was reported that VAT in premenopausal women may adversely affect the strength of the femoral neck bone, while the amount of subcutaneous fat mass in postmenopausal women deteriorates both femoral BMD and geometric indices of femoral neck strength.

There are numerous studies in the literature that have been performed to determine the relationship between BMD and lipids, and the findings reported by those studies are controversial. In a study performed by Yamaguchi et al. 11 with 214 patients between 47 and 86 years of age with postmenopausal vertebral fractures, a negative relationship between LDL-cholesterol and BMD ( *p* < 0.001) was revealed, as well as a positive association between not only HDL-cholesterol and TG, but also HDL-cholesterol and BMD ( *p* < 0.005 and *p* < 0.005, respectively). In a study by Cui et al. 15 performed on 375 premenopausal and 355 postmenopausal patients to demonstrate the relationship between BMD and the lipid profile, a significant negative relationship was reported between both total and BMD levels, and LDL-cholesterol and BMD levels in postmenopausal patients ( *p* < 0.001 and *p* < 0.001, respectively), and a positive relationship was also stated between BMD and TG levels ( *p* = 0.005). Even so, in another study conducted on 108 Turkish women by Sivas et al., 19 no difference was reported in terms of any lipid parameters in patients with and without osteoporosis. In our study, however, we found higher TG levels in women with osteoporosis, compared with those with normal BMD values; additionally, the levels of HDL-cholesterol were also found to be similar in all groups in our study.

As well as the use of anthropometric measurements such as BMI, WC, and waist-hip ratio to assess obesity, such factors as age, gender, hydration status, and ethnicity may also be influential on those measurements. It is known that increased BMI levels may be witnessed in an individual with high muscle mass; WC may be enhanced in cases where subcutaneous adipose tissue is high, making those anthropometric measurements inadequate in the evaluation of obesity, especially visceral obesity. 27 28 Computerized tomography (CT) and DXA used in the assessment of visceral obesity cannot be utilized in routine practice due to high cost, loss of time, and radiation risk. 29 30 As known, the level of VAI is calculated with a formula using BMI, WC, HDL-cholesterol, and TG levels. Considering the VAI formula, while an increase in the levels of WC and TG leads to an increase in the VAI levels, the increase in BMI and HDL-cholesterol levels are certain to cause a decrease in the VAI levels. In the literature, no study has investigated the relationship between BMD and VAI levels. In our study, there was no difference between the groups in terms of BMI and HDL-cholesterol levels. The levels of WC were found to be significantly higher in women with normal BMD values, compared with those with osteopenia and osteoporosis, and also higher in those with osteopenia, compared with those with osteoporosis; in addition, the levels of TG were also determined to be higher in women with normal BMD values, compared with those with osteoporosis. However, when the levels of VAI were calculated based on the formula using these two parameters, the difference was significant only between those with normal BMD values and the osteoporotic group, although the VAI levels were determined to be higher in those with normal BMD values than those in both osteopenic and osteoporotic patients, and also higher in the osteopenic patients than in those with osteoporosis. Given the readings of the correlation analysis, it was seen that both VAI and WC levels were positively correlated with the BMD lumbar spine *T* -score values; in other words, these levels were found to be somewhat protective of the development of osteoporosis. The levels of WC, which is an indicator of visceral adiposity, were found to be higher in those with normal BMD values, compared with those with osteopenia and osteoporosis; in addition, the WC levels were also detected to be higher in those with osteopenia, compared with those with osteoporosis. While the levels of VAI were determined to be higher only in those with normal BMI values than those with osteoporosis, no difference was witnessed between those with normal BMI values and osteopenia, and the groups with osteopenia and osteoporosis, suggesting that as the VAI levels decrease, the severity of the loss of BMD scores increases.

As mentioned in previous lines, the measurements of DXA can be used in the analysis of body fat distribution. 30 However, in our study we aimed to investigate the relationship between the VAI levels calculated by the formula and the DXA lumbar spine *T* -scores. Therefore, no analysis was performed to measure the amount of visceral fat through DXA, which is a limitation of our study. As another limitation, the nutritional habits and exercise status of our cases were questioned in our study. Adequate nutrition in terms of calcium and vitamin D is known to be protective against or to yield positive results in the treatment of osteoporosis 31 ; additionally, one study also reports similar and positive effects of regular physical exercise. 32 Even so, there are also conflicting studies stating the effects of nutrition with a high-protein diet on bone resorption and calcium excretion. 33 34

The effects of hyperinsulinemia and increased IR on bones are contradictory. As such, there are various studies reporting the positive effects of increased IR on BMD, 35 while there are also contradictory studies stating the adverse effects of increased IR on BMD. 36 In our study, the levels of HOMA-IR calculated in patients with normal BMD values, osteopenia, and osteoporosis were 3.2, 3.7, and 2.9, respectively, which were not so high. Moreover, no difference was detected in the between-group comparisons, and there was no correlation between the levels of HOMA-IR and VAI.

## Conclusion

In conclusion, the VAI levels used as an indicator of VAT were found to be higher in the group with normal BMD values, compared with those with osteoporosis. We also determined that WC and VAI levels had positive protective effects on the levels of the lumbar spine *T* -score. Waist circumference was found to be higher both in those with normal BMD values compared with those with both osteopenia and osteoporosis and in patients with osteopenia compared with those with osteoporosis; however, the fact that the difference was determined only between those with normal BMD values and osteoporosis in terms of VAI levels suggests that further studies including larger populations are needed to enlighten the entity.
